# Cannabis use and dependence among festival attendees: results from the French OCTOPUS survey

**DOI:** 10.1186/s12889-024-18496-9

**Published:** 2024-04-09

**Authors:** Sarah Chaaban, Marion Istvan, Benoit Schreck, Pauline Laigo, Morgane Rousselet, Marie Grall-Bronnec, Stéphanie Pain, Caroline Victorri-Vigneau

**Affiliations:** 1 Centre d’Evaluation et d’Information sur la Pharmacodépendance-Addictovigilance (CEIP-A), Service de Pharmacologie Clinique, Nantes Université, CHU Nantes, F-44000 Nantes, France; 2 Nantes Université, Univ Tours, CHU Nantes, CHU Tours, INSERM, MethodS in Patients-centered outcomes and HEalth Research, SPHERE, F-44000 Nantes, France; 3 UIC Psychiatrie et Santé Mentale, Nantes Université, CHU Nantes, F-44000 Nantes, France; 4 Centre d’addictovigilance, Service de Pharmacologie Clinique, CHU de Poitiers, 86000 Poitiers, France; 5 Laboratoire de Neurosciences Expérimentales et Cliniques, INSERM U-1084, Université de Poitiers, 86000 Poitiers, France

**Keywords:** Cannabis, Festival attendees, Cannabis dependence, DSM-IV criteria, Addictovigilance, Party scene

## Abstract

**Background:**

Chronic use of cannabis is associated with an increased risk of psychosocial, mental and physical health impairments. Sociohealth institutions reach a very limited proportion of cannabis users in need of treatment. Using data collected from festival attendees, this study aimed to estimate the prevalence of dependent cannabis users and to characterize cannabis dependence.

**Methods:**

We used data from the cross-sectional OCTOPUS survey carried out at 13 music events in the French department of Loire-Atlantique between July 2017 and July 2018. 383 participants aged 18 or older underwent a face-to-face interview about their basic sociodemographics, tobacco use, alcohol use and past-year substance use. Using the Diagnostic and Statistical Manual of Mental Disorders, 4th Edition (DSM-IV) criteria, we estimated the prevalence of dependent cannabis users and characterized their dependence.

**Results:**

More than two-thirds of participants reported that they had used cannabis in the past 12 months. Among 194 regular cannabis users (at least monthly), 63.4% were dependent. At least 40% of regular users reported health and/or social consequences of cannabis use. Compared to nondependent cannabis users, dependent cannabis users were more likely to be stimulant users and hallucinogen users.

**Conclusions:**

Dependent cannabis use is common among festival attendees, especially among stimulant or hallucinogen users. Festival settings may be important arenas for i) implementing efficient harm reduction measures to prevent dependence and ii) providing information on care structures and promoting the use of care to dependent users. In addition, healthcare professionals should be aware of trends in polysubstance use among dependent cannabis users.

**Supplementary Information:**

The online version contains supplementary material available at 10.1186/s12889-024-18496-9.

## Introduction

### Cannabis: a widespread substance leading to substantial health impairments

Cannabis is a plant that has been cultivated and used for recreational and medicinal purposes for many centuries. Its major psychoactive component is Δ9-tetrahydrocannabinol (THC) [1]. THC interacts with the endocannabinoid system and acts as a partial agonist at cannabinoid receptors CB1 and CB2. In Europe, cannabis is currently the most widespread psychoactive substance used. The prevalence of cannabis use is estimated to be five times that of other psychoactive substances [2]. A recent report estimates the prevalence of European adults who have consumed cannabis in the past year to be around 8% and even 18.2% among the younger (15–24 years old) [3]. A non-negligible portion uses cannabis on a daily or near-daily basis. The authors note that the level of use and trends varies by country. Policies and regulation of cannabis detention and consumptions differs between European countries, but do not explain the difference in prevalence. For instance, cannabis is illegal in France, and yet, France has the highest prevalence of cannabis consumption in Europe followed by Spain and Denmark. According to the European Drug Report and the French Health Barometer, lifetime cannabis use increased from 44.8 to 47.3% in the French adult population (aged 15–64) between 2017 and 2021 [2, 4]. Moreover, despite a slight reduction compared to previous years, the prevalence of daily cannabis users in France remained relatively high in 2021 (1.7%) [4].

Cannabis is often perceived as a harmless psychoactive substance. This belief may partly be explained by the low risk perception that users have of the drug and its increased availability on the drug market [2, 5]. Nevertheless, cannabis consumers can experience adverse consequences. The European report of 2023 states that cannabis is reported to be responsible for almost a third of all drug treatment admissions in Europe [3]. Short-term side effects of cannabis use are associated with psychiatric effects (such as anxiety, disorientation and derealization) and/or physical impairments (such as dry mouth, increased blood pressure and tachycardia) [6]. In general, these disorders are well tolerated, and intoxication remains non serious [7–9]. In contrast, chronic use of cannabis may frequently lead to substantial health impairments, including somatic disorders, cognitive and psychiatric dysfunctions or even cannabis abuse or dependence [6, 10]. Concerning somatic disorders, the most severe and documented risks are effects on cardiovascular system including tachycardia, coronary syndrome, strokes, arteritis, cardiomyopathies sometimes even for occasional use of cannabis [11–14]. Concerning psychiatric disorders: amotivational syndrome (lack of motivation, interest or enthusiasm in pursuing and engaging in activities) is associated with long-term cannabis use [6]; involvement of cannabis is suspected in schizophrenia [15] and cannabis-induced psychosis [16]. Concerning risk of dependence, 22.1 million people worldwide meeting the Diagnostic and Statistical Manual of Mental Disorders, 4th Edition (DSM-IV) diagnostic criteria making cannabis dependence the most prevalent drug use disorder according to the Global Burden of Diseases, Injuries and Risk Factors Study (GBD) 2016 [17].

### Cannabis use disorders: a public health concern

Worldwide, extensive epidemiological surveys have been conducted to estimate the prevalence of cannabis abuse or dependence in the general population. The results from these studies have been inconsistent, in large measure as a result of methodological differences, use of various cannabis use disorder screening tools, cultural variation, year of data collection or THC content [18–21]. For example, in a systematic review on the prevalence of cannabis use disorders (CUDs), the diagnosis of dependence varied from 3.5 to 40.9%, and the pooled estimated prevalence of cannabis dependence was 13% [18]. In France, the most recent data on cannabis use disorders are from the 2017 French Health Barometer, which showed that 25% of past-year cannabis users were at high risk of problematic use or dependence [22]. In festival settings, data on cannabis use disorders are scarce. One Danish study estimated the prevalence of cannabis dependence among party attendees and showed that out of 143 past-year cannabis users, 15% screened positive for cannabis dependence according to the Severity of Dependence Scale [23]. Due to the high prevalence of cannabis use, more research is needed to assess the risks associated with its consumption, especially since high-THC content products, which are known to be associated with higher rates of dependency, have become widely disseminated [2].

### Festival attendees: a population of young consumers

The regulars of urban events, like music festivals, has allowed a diversification of psychoactive substances available at urban music festival [24]. Substances reported to be used in festival and music events include mainly cannabis, stimulants such as MDMA/ecstasy or cocaine, and hallucinogens such as LSD, mushrooms or ketamine [25–27]. With its recreational properties, cannabis has been found to be particularly popular among party attendees, with a high prevalence of use regardless of the country or the type of festival or musicevents [28, 29]. Different factors have been associated to psychoactive substances consumptions such as age, sex, lifestyle, employment and also festivals and their music style [30]. Stimulants, LSD or cannabis have been the most reported psychoactive substances in techno scene [25, 31–33]. Other styles of music are emerging in dedicated festivals, such as the Dub, reggae’s subgenre. There are very few studies which have been conducted about consumption of psychoactive substances at a reggae festival and even less so in dub music festivals [34], so cannabis consumption in this kind of music festival is unknown. Palamar et al. found that American students who had attended a rave party had a higher prevalence of past-year cannabis use (56.3%) than non-attendees (31.7%) [35]. Feltmann et al. interviewed frequent attendees of electronic dance music events and found that 51% of participants had consumed cannabis during the past year [36]. Another study among 1365 subjects conducted at a music festival in Australia demonstrated that 84% of the party attendees had used cannabis in the past month [37].

This population is all the more interesting because it is a population of young consumers who do not seek care [38]: they are generally young and heathy, thus not iunder medical supervision. It is therefore very difficult to evaluate consumption and its consequences. The lack of knowledge about the negative consequences with drug use in this population have also been highlighted by Feltman et al. [36]. The OCTOPUS survey [39] was designed by the French regional addictovigilance center of Pays de la Loire to improve knowledge on illicit drug use by collecting data on party attendees’ consumption. The main purpose of this work was to estimate the prevalence of dependent cannabis users and to characterize cannabis dependence among festival attendees using data from the OCTOPUS survey.

## Materials and methods

### Procedure

OCTOPUS is a cross-sectional survey set up by the French regional CEIP-A of Pays de la Loire and funded by the Regional Health Agency of Pays de la Loire. Addictovigilance is defined as a specific French health vigilance method intended to monitor medicinal or illicit psychoactive substance abuse or dependence and is composed of 13 drug dependence evaluation and information centres (Centres d’Evaluation et d’Information sur la Pharmacodépendance - Addictovigilance - CEIP-A). Addictovigilance fulfils three main roles: (i) to collect and assess cases of problematic drug consumption (through the mandatory reports of health professionals and multiple epidemiological tools related to drug use disorders), (ii) to inform healthcare professionals about potential substance dependence and (iii) to conduct research [40]. Consequently, addictovigilance contributes to the protection of public health by providing information and implementing harm reduction measures [41].

The survey was monitored by a multidisciplinary committee of pharmacologists specialized in addictology and social workers involved in harm reduction measures associated with drug use and management of addiction prevention or treatment.

### Participants

Participants were randomly recruited from 13 various music festivals (electronic, dub and eclectic music) taking place in the French department of Loire-Atlantique between July 2017 and July 2018. The inclusion criteria were being at least 18 years of age, being present at the site of the event and understanding the questionnaire. The festivals where the participants were recruited were emblematic of the region: there is a large dub festival that is among the largest in Europe, and the electronic music scene is also well represented in the regional music landscape.

### Data collection

Data collection was undertaken by trained volunteer interviewers at music festival sites before 1:00 a.m. using face-to-face interviews. The data collected included sociodemographic characteristics, past-year substance use (tobacco, alcohol and illicit substances) and harm reduction knowledge.

In this work, we focused on past-year cannabis users (i.e., use of cannabis at least once in the previous 12 months), and we analysed the following variables:

- The music style of the festival;
- Sociodemographic data (sex, age, region of residence, employment status, household type and education);
- The presence of long-term disease(s) and long-term medication treatment(s) [42];
- The frequency of tobacco use, alcohol use and binge drinking (i.e., 6 or more drinks on one occasion); and
- Past-year illicit drug use (classified into one of the following subgroups: stimulants, hallucinogens, sedatives or new psychoactive substances (NPSs)) and characteristics of illicit drug use (name, setting of use, type, route of administration, frequency of consumption, anteriority of consumption, last consumption, mode of obtaining, effects sought, adverse events and DSM-IV criteria for dependence).

### Ethics

The study was conducted in accordance with the 1964 Helsinki declaration and its later amendments. Informed consent was collected from each participant during the interview. According to French regulation, ethical approval was not required as the survey is not within the scope of application of the French Public Health Code, according to both articles R1121-1 and L1123-6 [43, 44]. Indeed, for the OCTOPUS survey, only anonymous data in the context of the regulated purposes of the addictovigilance system were collected.

### Outcomes

The primary outcome was the prevalence of cannabis dependence among regular users and the characteristics of dependence.

Dependence was assessed among regular users, i.e., participants who had used cannabis at least monthly in the last 12 months.

According to the DSM-IV, dependence is defined by at least three positive dependence criteria from 0 to 7 using the following items: tolerance; withdrawal; the substance is taken in larger amounts or over a longer period than intended; persistent desire or unsuccessful efforts to cut down or control substance use; great deal of time is spent in activities necessary to obtain, use the substance or recover from its effects; important social, occupational or recreational activities are given up or reduced because of substance use; and the substance is continued despite the knowledge of having a persistent or recurrent physical or psychological problem that is likely to have been caused or exacerbated by the substance.

### Statistical analysis

Descriptive statistics are expressed as numbers and percentages for categorical variables. The prevalence of dependent cannabis users was calculated as the number of cannabis users with a dependence out of the number of regular cannabis users and out of the total number of cannabis users. Fisher’s exact test or the chi-square test was used to compare the characteristics between dependent cannabis users and nondependent cannabis users.

Statistical analyses were performed using SAS® software version 9.4 (SAS Institute Inc).

## Results

### General description

A total of 243 past-year cannabis users (63.4%) were included in our work out of the 383 subjects of the OCTOPUS survey.

#### Characteristics of cannabis users

The characteristics of past-year cannabis users are presented in Table 1. The majority of them (45.7%) were recruited at electronic music festivals, 33.7% at eclectic music festivals and 20.6% at dub music festivals. Past-year cannabis users were mainly males (68%) and under 30 years of age (73%). Nearly three-quarters (71.1%) were from the Pays de la Loire region. More than half of cannabis users were employed (58.8%), 28.8% were students, and 9.7% were unemployed. Few cannabis users reported comorbidities (12.4%) or regular medication treatment (9.6%).

**Table 1:** Characteristics of past-year cannabis users (*N* = 243 subjects)

		*N* = 243
**Sociodemographic data**	**Music style of the festival,** ***n*** **(%)**	
	Electronic	111 (45.7)
	Eclectic	82 (33.7)
	Dub	50 (20.6)
	**Sex,** ***n*** **(%)** ^**(1)**^	
	Male	165 (68.2)
	Female	77 (31.8)
	**Age (years),** ***n*** **(%)**	
	18–24	100 (41.1)
	25–29	78 (32.1)
	≥ 30	65 (26.8)
	**Region of residence,** ***n*** **(%)** ^**(1)**^	
	Pays de la Loire	172 (71.1)
	Bretagne	24 (9.9)
	Other regions	46 (19.0)
	**Employment status,** ***n*** **(%)** ^**(2)**^	
	Employed	140 (58.8)
	Student	67 (28.2)
	Unemployed	23 (9.7)
	Volunteer/civic service	8 (3.3)
	**Education,** ***n*** **(%)** ^**(1)**^	
	Higher education	150 (62.0)
	High school/baccalaureate diploma	64 (26.4)
	First stage of primary or secondary education	28 (11.6)
	**Household type,** ***n*** **(%)** ^**(2)**^	
	Living alone	104 (44.4)
	Living as a couple	57 (24.4)
	Living with other people	43 (18.4)
	Living with parents	30 (12.8)
**Health data**	**Long-term disease,** ***n*** **(%)** ^**(1)**^	
	Yes	30 (12.4)
	No	212 (87.6)
	**Long-term medication treatment,** ***n*** **(%)** ^**(2)**^	
	Yes	23 (9.6)
	No	216 (90.4)
**Consumption data**	**Tobacco use,** ***n*** **(%)** ^**(1)**^	
	Festive	24 (9.9)
	Yes, frequency unknown or < 5 cigarettes per day	38 (15.7)
	5 to 10 cigarettes per day	84 (34.7)
	> 10 cigarettes per day	71 (29.4)
	No	25 (10.3)
	**Alcohol use,** ***n*** **(%)**	
	Festive	96 (39.5)
	Yes, frequency unknown or < 1 glass per day	68 (28.0)
	Yes, > 1 glass per day	74 (30.4)
	No	5 (2.1)
	Binge drinking*, number of times per month ^**(2)**^	
	≤ 1 time	63 (26.4)
	2 to 4 times	85 (35.6)
	> 4 times	72 (30.1)
	Yes, frequency unknown	19 (7.9)
	**Stimulant use,** ***n*** **(%)**	
	Yes	166 (68.3)
	No	77 (31.7)
	**Hallucinogen use,** ***n*** **(%)**	
	Yes	83 (34.2)
	No	160 (65.8)
	**Sedative use,** ***n*** **(%)**	
	Yes	14 (5.8)
	No	229 (94.2)
	NPS** use, ***n*** **(%)**	
	Yes	14 (5.8)
	No	229 (94.2)

* Consumption of 6 or more drinks of alcohol during a single occasion

** NPS: New psychoactive substance

Missing data: (1): < 1%, (2): 1 to 5%

Almost all of the cannabis users reported tobacco and alcohol use (90 and 98%, respectively), and a third reported binge drinking at least four times per month. Cannabis users also consumed stimulants (68.3%), hallucinogens (34.2%), sedatives (5.8%) and NPSs (5.8%). Cocaine and ecstasy/methylenedioxymethamphetamine (MDMA) were the most commonly used illicit substances among cannabis users (55.1 and 45.3%, respectively) (Supplementary material Table S1).

#### Characteristics of cannabis consumption

Table 2 displays the characteristics of past-year cannabis consumption of the 243 past-year cannabis users. A total of 258 occurrences of past-year cannabis consumption were reported by the respondents. There were more cannabis consumption events than cannabis users: indeed, 15 participants reported differences in cannabis consumption characteristics. For each one, only two consumption events were reported. Out of the 15, 12 participants reported consumption through different routes of administration (oral and inhalation). 3 participants reported two different type of cannabis (cannabis oil /cannabis herb; cannabis herb/ cannabis resin).

**Table 2:** Characteristics of past-year cannabis consumption (*N* = 258 consumption events; *N* = 243 subjects)

	*N* = 258
**Cannabis type,** ***n*** **(%)**	
Cannabis herb	163 (79.5)
Cannabis resin	41 (20.0)
Cannabis oil	1 (0.5)
**Route of administration,** ***n*** **(%)** ^**(1)**^	
Inhalation	232 (90.6)
Oral	24 (9.4)
**Frequency of consumption,** ***n*** **(%)** ^**(3)**^	
Experimentation	1 (0.4)
Daily	130 (55.8)
Weekly	53 (22.8)
Monthly	32 (13.7)
Quarterly	4 (1.7)
Annually	13 (5.6)
**Anteriority of consumption,** ***n*** **(%)** ^**(2)**^	
≤ 1 year	3 (1.2)
1 to 3 years	5 (2.0)
3 to 5 years	29 (11.4)
5 to 10 years	88 (34.6)
≥ 10 years	129 (50.8)
**Last consumption,** ***n*** **(%)** ^**(1)**^	
< 48 hours	201 (78.5)
≤ 1 week	18 (7.0)
≤ 1 month	20 (7.8)
< 1 year	17 (6.7)
**Mode of obtaining,** ***n*** **(%)** ^**(3)**^	
Friends	116 (47.9)
Dealer	67 (27.7)
Purchased without any information	17 (7.0)
Culture	16 (6.6)
Donation	11 (4.5)
Others	49 (20.2)
**Effect(s) sought,** ***n*** **(%)** ^**(2)**^	
Calming	194 (76.1)
Euphoria	56 (22.0)
Lettinggo	17 (6.7)
Clairvoyancy	14 (5.5)
Empathogenic	10 (3.9)
Stimulation	7 (2.7)
Getting high	5 (2.0)
Derealization	4 (1.6)
Chemsex	1 (0.4)
Other effects	21 (8.2)
**Cannabis adverse event(s),** ***n*** **(%)** ^**(3)**^	
Psychological	67 (31.3)
Somatic	65 (30.4)
Asthenia	25 (11.7)
Badtrip	9 (4.2)
Isolation	6 (2.8)
Cravings	3 (1.4)
Other symptoms	21 (9.8)

Missing data: (1): < 1%, (2): 1 to 5%, (3): > 5%

Cannabis herb (79.5%) and cannabis resin (20%) were the most common cannabis types consumed. Almost all cannabis consumption (90.6%) was by inhalation, while the oral route was less prevalent (9.4%). More than half of cannabis consumption occurred daily (55.8%), and 78.5% occurred within the last 48 hours preceding the festivals. Two hundred and fifteen cannabis consumption occurred at least monthly and therefore were considered regular [45]. Cannabis was mainly obtained through friends (47.9%) and dealers (27.7%). The most frequently sought sensations were “calming” (76.1%) and “euphoria” (22%). Psychological and somatic adverse events occurred in 31.3 and 30.4% of cannabis consumption events, respectively.

### Cannabis dependence

#### Prevalence of cannabis dependence

The 215 regular consumption events corresponded to 203 participants (as indicated above one participant could have two consumption events). Due to missing data dependence could be assessed for 194 regular cannabis users. The prevalence of 3 or more positive DSM-IV dependence criteria was 63.4% among regular users (123/194), corresponding to 50.6% of total users (123/243).

**Fig. 1:**
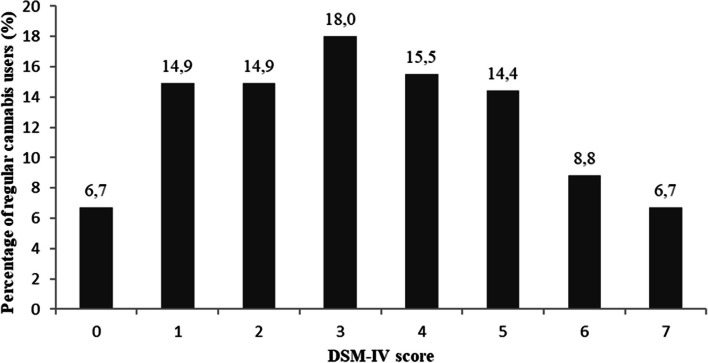
Distribution of DSM-IV dependence scores for regular cannabis users within the last 12 months (*N* = 194)

Figure 1 presents the quantitative distribution of DSM-IV dependence criteria for regular cannabis users (*n* = 194). As indicated above, 63.4% of regular cannabis users had a DSM-IV score greater than or equal to 3, and 47.9% had a DSM-IV score between 3 and 5. The proportion of cusers with a score of 6 or 7 ranged from 6.7 to 8.8%.

#### Characteristics of dependence

**Fig. 2:**
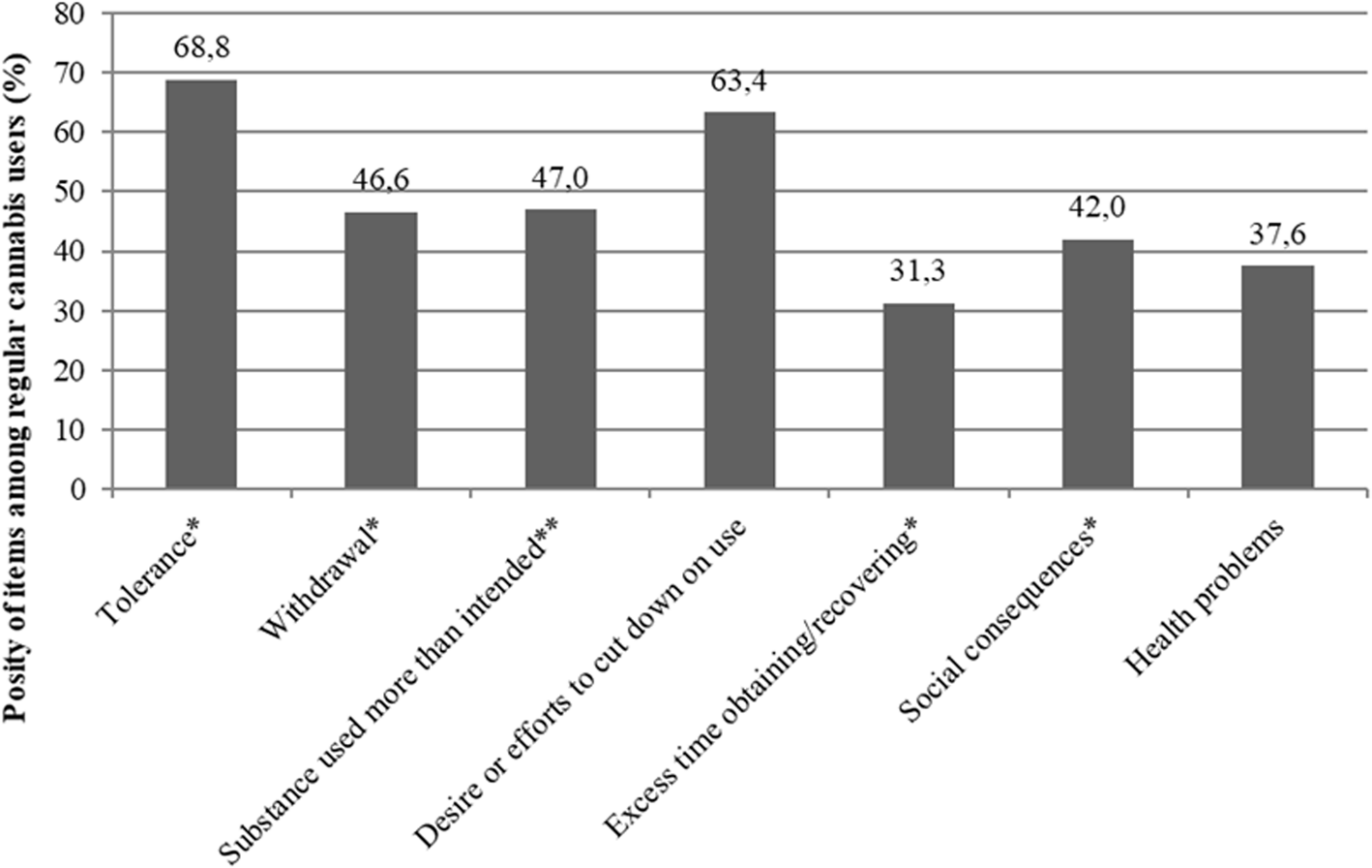
Proportion of positivity of DSM-IV criteria for dependence (*N* = 194 users)

The frequency of positivity for each DSM-IV criterion for dependence is presented in Fig. 2. The most highly endorsed DSM-IV criteria were “tolerance” (68.8%) and “desire or efforts to cut down on use” (63.4%), followed by “substance used more than intended” (47.0%) and “withdrawal” (46.6%) (Fig. 2). Social consequences and health problems were reported for 42.0 and 37.6% of cannabis users, respectively. The least endorsed DSM-IV criterion was “excess time obtaining/using the substance” (31.3%).

### Characterization of dependent cannabis users

Differences between dependent cannabis users and nondependent cannabis users are summarized in Table 3. Compared to nondependent cannabis users, dependent cannabis users were more likely to be stimulant users (*p* = 0.0003) and hallucinogen users (*p* = 0.014). The percentage of cannabis-dependent users seemed higher among participants who attended electronic and dub music festivals, but the *p*-value was not significant (0.094). (*p* = 0.094).

**Table 3:** Comparison of characteristics between dependent and nondependent regular cannabis users (*N* = 194 regular users)

	Regular cannabis users (*N* = 194)		
Dependence	Yes (*n* = 123)	No (*n* = 71)	*P*
**Music style of the festival,** ***n*** **(%)**			
Electronic	59 (48.0)	28 (39.4)	0.094
Eclectic	35 (28.5)	31 (43.7)	
Dub	29 (23.5)	12 (16.9)	
**Sex,** ***n*** **(%)**			0.293
Male	83 (67.5)	53 (74.7)	
Female	40 (32.5)	18 (25.3)	
**Age (years),** ***n*** **(%)**			0.160
18–24	55 (44.7)	25 (35.2)	
25–29	39 (31.7)	24 (33.8)	
≥ 30	29 (23.6)	22 (31.0)	
**Region of residence,** ***n*** **(%)** ^**(1)**^			0.743
Pays de la Loire	90 (73.8)	54 (76.1)	
Bretagne	10 (8.2)	7 (9.8)	
Other regions	22 (18.0)	10 (14.1)	
**Employment status,** ***n*** **(%)** ^**(2)**^			0.540
Student	34 (28.6)	18 (25.4)	
Unemployed	13 (10.9)	5 (7.0)	
Employed	72 (60.5)	48 (67.6)	
**Education,** ***n*** **(%)** ^**(1)**^			0.894
Higher education	73 (59.8)	41 (57.7)	
High school/baccalaureate diploma	34 (27.9)	22 (31.0)	
First stage of primary or secondary education	15 (12.3)	8 (11.3)	
**Household type,** ***n*** **(%)** ^**(2)**^			0.314
Living alone	53 (45.3)	26 (38.2)	
Living as a couple	22 (18.8)	21 (30.9)	
Living with other people	27 (23.1)	14 (20.6)	
Living with parents	15 (12.8)	7 (10.3)	
**Long-term disease,** ***n*** **(%)**			0.513
Yes	16 (13.0)	7 (9.9)	
No	107 (87.0)	64 (90.1)	
**Long-term medication treatment,** ***n*** **(%)** ^**(1)**^			0.846
Yes	11 (9.0)	7 (9.9)	
No	111 (91.0)	64 (90.1)	
**Tobacco use,** ***n*** **(%)** ^**(1)**^			0.541
Yes	110 (90.2)	62 (87.3)	
No	12 (9.8)	9 (12.7)	
**Alcohol use,** ***n*** **(%)**			0.358
Yes	121 (98.4)	68 (95.8)	
No	2 (1.6)	3 (4.2)	
Binge drinking***, number of times per month ^**(2)**^			
≤ 1 time	30 (24.8)	21 (30.0)	0.504
2 to 4 times	42 (34.7)	28 (40.0)	
> 4 times	37 (30.6)	17 (24.3)	
Any	12 (9.9)	4 (5.7)	
**Use of at least one stimulant,** ***n*** **(%)**			0.0003
Yes	95 (77.2)	37 (52.1)	
No	28 (22.8)	34 (47.9)	
**Use of at least one hallucinogen,** ***n*** **(%)**			0.014
Yes	53 (43.1)	18 (25.3)	
No	70 (56.9)	53 (74.7)	
**Use of at least one sedative,** ***n*** **(%)**			1.000
Yes	7 (5.7)	5 (5.6)	
No	116 (94.3)	67 (94.4)	
**Use of at least one NPS,** ***n*** **(%)**			1.000
Yes	8 (6.5)	4 (5.6)	
No	115 (93.5)	67 (94.4)	

*** Consumption of 6 or more alcoholic drinks during a single occasion

Missing data: (1) < 1%, (2): 1 to 5%

## Discussion

### Review of major findings

In this study, we used data from the OCTOPUS survey to estimate the prevalence of dependent cannabis users and characterize dependent cannabis use among festival attendees.

Our results demonstrated high numbers of past-year cannabis users (63.4%) in the sample of party attendees. According to the DSM-IV criteria, almost two-thirds of the 194 regular cannabis users screened positive for dependence. Most previous studies reviewed found a lower prevalence of cannabis dependence in large representative samples of the general population [18, 46, 47] or in festival settings [23]. In contrast, only one study among nearly 35,000 Canadian participants found that 82.5% of past-month cannabis users were problematic users as determined by the Alcohol, Smoking, and Substance Involvement Screening tool [48]. These discrepancies may be due to methodological differences, such as the use of various cannabis use disorder screening tools, year of data collection or THC content.

In our comparative analysis, factors associated with cannabis dependence included past-year use of stimulants or hallucinogens. Our findings are consistent with those in the literature in which polysubstance use among cannabis consumers increased the likelihood of cannabis dependence [49–51]. Different theories have been proposed to explain these correlations. From a pharmacological view, cannabis may be used to manage the unpleasant effects induced by the use of other substances [49, 52]. From a psychosocial view, gateway drug theory suggests that lifetime cannabis use may increase exposure to other illicit substances [52, 53]. Other reasons that could partly explain these associations include greater accessibility of illicit drugs, personality traits and social determinants [49, 50].

We found that the most prevalent DSM-IV criteria for cannabis dependence were related to physical dependence (i.e., “tolerance” and “withdrawal”). Based on a recent meta-analysis of observational studies, cannabis withdrawal syndrome (CWS) appears to be common and concerns nearly half of regular or dependent cannabis users [54]. Similarly, “tolerance” was found to be one of the most highly reported criteria [55–57]. One reason that could probably explain these high rates is that tolerance and CWS are physiologic responses to chronic use regardless of sociodemographic and environmental determinants [57]. Regarding social and health consequences, we found that nearly 40% of regular cannabis users reported such consequences, which was higher than those in other previous studies [55, 57]. However, these discrepancies are not surprising regarding the heterogeneity in the study design, and a multitude of external factors, such as social environment, personal motivations or underlying diseases, can be associated with the appearance of negative consequences.

### DSM-IV versus DSM-5 criteria

In this study, we used the DSM-IV criteria for the assessment of cannabis dependence, although the DSM-5 criteria have already been published. The DSM-IV separates diagnoses of substance abuse and substance dependence using a checklist of seven criteria for the latter. One of the most notable changes in the DSM-5 concerns the combination of substance abuse and substance dependence diagnoses to create a single category of substance use disorder triggered by any two or more of 11 criteria, with six or more indicating a severe case [58]. Few studies have assessed the concordance between DSM-IV and DSM-5 for dependence diagnosis and found that correspondence was excellent, even though the psychometric properties for cannabis were not as good as for other substances [59–61]. In our opinion, the DSM-IV is better suited to measure cannabis dependence in festival settings since it has fewer criteria than the DSM-5 and thus is quicker to conduct.

According to the DSM-IV criteria, almost two-thirds of the 194 regular cannabis users scored 3 or above and thus screened positive for dependence. With the use of the DSM-5, we would expect a higher prevalence, as substance use disorder is defined by at least two positive criteria. Indeed, in our study, 77% of cannabis consumption events corresponded to a DSM-IV score of at least 2.

### Study implications

In France, the monitoring of abuse and dependence related to psychoactive substances is mainly based on spontaneous reporting by healthcare professionals. According to the last national epidemiological survey exploring the adverse events of recreational cannabis use reported to the French addictovigilance network between 2012 and 2017 (*n* = 2217), the most common effect was dependence, ranging from 10.1 to 20.3% over the study period. Men were mostly involved (76%), and a history of substance abuse (stimulants, opioid drugs, hallucinogens and depressant drugs) was reported in 35% of cases [62]. Nevertheless, a limitation of this health surveillance system is that it only includes reports of individuals who attend sociohealth institutions. Recreational drug users constitute a hard-to-reach population for the French addictovigilance network [63–65] because they rarely attend sociohealth institutions except in cases of acute intoxication. However, we found in our study that at least 40% of regular users reported negative consequences, such as social or health consequences, reflecting the need for care and support.

### Harm reduction strategies

In general, strategies for preventing the adverse effects of chronic cannabis use are based on school and community substance use prevention programs [66, 67]. The target population is mainly young people, as evidence suggests that early initiation of cannabis use during adolescence might precipitate negative outcomes in adulthood [68]. In light of our findings, festival settings may be important arenas for i) implementing efficient harm reduction measures to prevent dependence and ii) providing information on care structures and promoting the use of care for dependent users. It is essential to communicate the possible negative consequences of chronic cannabis use, as this use is often trivialized.

In addition, healthcare professionals should be aware of trends in polysubstance use among problematic cannabis users since it may increase the risk of negative health-related consequences. However, as many users consume cannabis at home, there is a need to find other ways to target cannabis users and inform them of the possible chronic risks. Digital interventions such as addiction prevention websites, chat-based interventions, and mobile applications could be promising tools for ensuring a broader population health approach and reducing problematic cannabis use [65].

### Strengths and limitations

The main strength of our study was the access to a “hidden population” of cannabis users, and to our knowledge, no similar study has been undertaken recently in France. In addition, data collection was standardized, as all the interviewers were trained in the data collection tool. Festival attendees were recruited from many different music events but restricted to one geographic area (Pays de la Loire region), which prevents us from generalizing our results. However, according to the last data of the French Health Barometer, past-year cannabis use in the French region of Pays de la Loire was quite similar to the national level (10.2 and 11%, respectively) [4]. Moreover, well-known limitations of self-reported data collection could also be underlined, such as social desirability bias or recall bias. However, we think that the status of the interviewers (volunteers trained in addiction care or harm reduction strategies) may have reduced the likelihood of inaccurate reporting.

## Conclusion

In conclusion, cannabis dependence was frequent in our study among festival attendees, and we also highlighted a high rate of regular users reporting social or health consequences. We also found an association between the use of stimulants or hallucinogens and cannabis use dependence. In light of our findings, festival settings may be important arenas for implementing efficient harm reduction measures and preventing the development of dependence among cannabis users. In addition, healthcare professionals should be aware of trends in polysubstance use among dependent cannabis users.

### Supplementary Information

**Supplementary Material 1.**

## Data Availability

No datasets were generated or analysed during the current study.
