# Supplementary material for: Cannabis use and dependence among festival attendees: results from the French OCTOPUS survey
Source: BMC Public Health. 2024 Apr 9;24:992. doi: 10.1186/s12889-024-18496-9 (PMC11003156; doi:10.1186/s12889-024-18496-9)
Supplement: Supplementary file 1 — Supplementary Material 1. [file 12889_2024_18496_MOESM1_ESM.docx]

**Supplementary material**

**Table S1**. Prevalence of past-year illicit drug consumptions among cannabis users (N = 243 subjects).

|  | **N = 243** |
| --- | --- |
| **Stimulants, n (%)** |  |
| Ecstasy/MDMA* | 134 (55.1) |
| Cocaine | 110 (45.3) |
| Speed | 18 (7.4) |
| Poppers | 15 (6.2) |
| Amphetamines | 5 (2.1) |
| GBL** | 2 (0.8) |
| Ritaline | 1 (0.4) |
| Ephedrine | 1 (0.4) |
| Caffeine | 1 (0.4) |
| **Hallucinogens** |  |
| LSD*** | 47 (19.3) |
| Mushrooms | 32 (13.2) |
| Ketamine | 28 (11.5) |
| Salvia divinorum | 1 (0.4) |
| Mescaline | 1 (0.4) |
| **New Psychoactive Substances** |  |
| DMT | 3 (1.2) |
| Mephedrone | 3 (1.2) |
| U47700 | 2 (0.8) |
| Methoxetamine | 2 (0.8) |
| Synthetic cannabinoid | 2 (0.8) |
| 2C-B | 2 (0.8) |
| 5-APB | 1 (0.4) |
| Ethylphenidate | 1 (0.4) |
| 25C-NBOME | 1 (0.4) |
| 25I-NBOME | 1 (0.4) |
| 2C-E | 1 (0.4) |
| 2C-P | 1 (0.4) |
| Methylone | 1 (0.4) |
| **Sedatives** |  |
| Opium | 5 (2.1) |
| Heroin | 2 (0.8) |
| Nitrous oxide | 2 (0.8) |
| Tramadol | 2 (0.8) |
| Valium | 2 (0.8) |
| Methadone | 1 (0.4) |
| Codeine | 1 (0.4) |
| Skenan | 1 (0.4) |

**MDMA: Methylenedioxymethamphetamine, **GBL: gamma-butyrolactone, ***LSD: [Lysergicaciddiethylamide](https://en.wikipedia.org/wiki/Lysergic_acid_diethylamide)*
